# West Texas District Medical Society

**Published:** 1896-11

**Authors:** Jno. W. Kenney

**Affiliations:** Secretary W. T. M. Association; San Antonio, Texas


### West Texas District Medical Society—Annual Meeting.

San Antonio, Texas, October 30, 1896.

*Editors Texas Medical Journal:*

The following resolutions relative to a mixed examining board was read and endorsed by the Western Texas Medical Association at its annual meeting, held in this city, October 29, 1896:

"Whereas, A bill authorizing the appointment of a State examining board to be composed of regular physicians, homeopaths and eclectics, has been formulated and endorsed by the State Medical Association, and is to be presented by them to the next legislature for its enrollment into a law; therefore be it

"*Resolved*, By the Western Texas Medical Association, that we believe a board so constituted to be contrary to the Code of Ethics and Traditions of the regular profession and derogatory of its best interests."

The annual meeting just held has been a very interesting one and was largely attended by the members and their friends from all parts of the district.

The following programme constituted the day's work:

PROGRAMME.

Morning Session, 10:30 P. M.—Dr. F. Paschal—"Civilization and Tuberculosis."

Dr. J. H. Bell—"An Analytical study of the Ocular Muscles and the Fusion of Images, Their Intimate Relations to Heterotropia and Hypermetropia."

Afternoon Session, 2:30 P. M.—Dr. R. E. Moss--"Chronic Suppurative Otitis."

Dr. B. F. Kingsley—"Methods and Technique in Surgery."

Dr. S. Burg—"Pyothorax and its Relations to Subphrenic Abscess."

Evening Session, 8 P. M.—Regular business and election of officers.

President's annual address on Medical Mistakes by Dr. B. E. Hadra, San Antonio, Texas.

Banquet.

The following officers were elected for the ensuing year:

President, Dr. R. E. Moss, of San Antonio; 1st vice-president, Dr. A. C. McDaniel, of San Antonio; 2nd vice-president, T. T. Jackson, of San Antonio.

Board of Censors.—Dr. B. E. Hadra (chairman), of San Antonio; Dr. C. E. Keller, of Marion; Dr. B. F. Kingsley, of San Antonio; Dr. S. Burg, of San Antonio.

The past year has been an eventful one in the history of the Association. A nucleus for a pathological laboratory and a medical library has been established, and a more fraternal feeling brought about among the profession in this part of the State.

Very truly yours,

JNO. W. KENNEY, M. D.,
Secretary W. T. M. Association.

